# Bladder cancer cells re-educate TAMs through lactate shuttling in the microfluidic cancer microenvironment

**DOI:** 10.18632/oncotarget.5538

**Published:** 2015-10-13

**Authors:** Yang Zhao, Degui Wang, Ting Xu, Pengfei Liu, Yanwei Cao, Yonghua Wang, Xuecheng Yang, Xiaodong Xu, Xinsheng Wang, Haitao Niu

**Affiliations:** ^1^ Department of Surgery, Affiliated Hospital of Qingdao University, Qingdao, China; ^2^ Department of Anatomy, School of Basic Medical Sciences, Lanzhou University, Lanzhou, China; ^3^ Department of Geratology, The 401st Hospital of PLA, Qingdao, China; ^4^ Department of Urology, Affiliated Hospital of Qingdao University, Key Laboratory of Urinary System Diseases, Qingdao, China

**Keywords:** bladder cancer, lactate, microfluidic chips, tumor-associated macrophage, re-education

## Abstract

**Background:**

In the present study, we aimed to investigate the influence of lactate shuttling on the functional polarization and spatial distribution of transitional cell carcinoma of the bladder (TCCB) cells and macrophages.

**Methods:**

We designed a microfluidic coculture chip for real-time integrative assays. The effect of lactate shuttling on the re-education of macrophages by TCCB cells was explored by measuring the levels of NO using a total NO assay kit and by evaluating the protein expression of iNOS, p-NFkB-p65, Arg-1 and HIF-1α via cell immunofluorescence and western blotting. Additionally, we examined TCCB cell viability using acridine orange/ethidium bromide (AO/EB) and MitoTracker staining. Moreover, the concentration distributions of lactate and large signaling proteins in the culture chambers were measured using 4′,6-diamidino-2-phenylindole (DAPI) and fluorescein isothiocyanate-dextran (FITC-dextran). Furthermore, the recruitment of macrophages and the influence of macrophages on BC metastasis were observed via light microscopy.

**Results:**

We confirmed that TCCB cells reprogrammed macrophages into an M2 phenotype. Moreover, lactate inhibited M1 polarization and induced M2 polarization of macrophages, but blockade of cancer cell-macrophage lactate flux significantly inhibited the re-education of macrophages by TCCB cells. In addition, lactate diffused faster and deeper than large signaling proteins in the microfluidic tumor microenvironment. Furthermore, lactate alone induced the migration of macrophages, and M1, but not M2, macrophages reduced the motility of TCCB cells.

**Conclusions:**

TCCB cells reprogrammed macrophages into an M2 phenotype in a manner that depended on cancer cell-TAM lactate flux. Furthermore, the lactate shuttle may be a determinant of the density of TAMs in tumor tissue.

## INTRODUCTION

Bladder cancer (BC) is one of the most common malignancies worldwide, with approximately 73,000 newly diagnosed cases and 15,210 cancer-related deaths in 2013 in the USA [1]. Currently, cancer immunotherapies such as intravesical Bacillus Calmette-Guerin (BCG) therapy have prevalently been utilized for BC treatment [2]. Although immunotherapy remarkably improves the prognosis of patients with BC, 30–50% of BC patients have no response to immunotherapy, and 15% of BC patients show cancer progression after treatment [3]. Cancer cell-macrophage interactions in the bladder cancer microenvironment have consistently been the focus of BCG therapy and adaptive immunotherapy [4, 5]. Thus, further research into the resistance of TCCB to BCG therapy is necessary, and functional reprogramming and regional relocation of tumor-associated macrophages (TAMs) are the key areas of focus in this research [6].

TAMs are remarkably plastic cellular components in the tumor microenvironment. These cells exhibit two oppositely programmed phenotypes: a classically activated phenotype (M1), which is observed in early stage tumors, and an alternatively activated phenotype (M2), which is observed in tumors that have progressed [7]. According to their opposing roles in cancer, two different cancer cell-macrophage interaction patterns correspond to an inflammatory or an anti-inflammatory pathway. In the inflammatory pathway, TAMs are activated by a tumor antigen and are then reprogrammed into M1 macrophages, leading to enhanced humoral and cellular immune reactions and increased apoptosis of cancer cells due to the production of nitric oxide (NO) and inflammatory cytokines, including tumor necrosis factor-α (TNF-α), interleukin (IL)-1β, IL-6 and IL-23 [8]. Alternatively, in the anti-inflammatory pathway, TAMs exhibit the M2 phenotype due to the induction of the cancer cell-mediated secretion of cytokines such as macrophage colony-stimulating factor (M-CSF), IL-4/IL-13, and IL-10 [8]. And M2 macrophages promote the proliferation, invasion and metastasis of cancer cells by producing more anti-inflammatory and pro-angiogenic cytokines, such as transforming growth factor-β (TGF-β), vascular endothelial growth factor (VEGF), epidermal growth factor (EGF) and fibroblast growth factor (FGF) [8, 9]. The status of the cancer cell-macrophage interaction pattern indicates the extent of immunosuppression inside the tumor niche and is associated with the efficiency of immunotherapy and the survival of patients with TCCB [3, 10, 11]. During the development and progression of cancer, TAMs can be recruited by cancer cells via the secretion of chemokines such as CCLs. TAMs can promote matrix deposition and remodeling and can prepare a pro-metastatic microenvironment for cancer cells [12]. The recruitment of macrophages and the relocation of TCCB cells are of vital importance to the progression and metastasis of TCCB, and enhanced recruitment of TAMs is significantly associated with late clinical staging and poor overall survival (OS) [13].

In our previous proteomic study of TCCB, we found common alterations in glycolytic enzymes such as L-lactate dehydrogenase subunit A (LDHA) in TCCB cells, especially in advanced cancer cells [14]. Moreover, the expression of LDH5 is positively correlated with the density of CD163 (+) TAMs, but not CD68 (+) TAMs. These findings indicate that aerobic glycolysis, also referred to as the Warburg effect, may participate in the reprogramming and recruitment of TAMs. Lactate, the major metabolite of aerobic glycolysis, and MCTs, which are known to function as lactate/proton symporters, have been considered as key effectors of cancer metabolism that play an important role in the prediction of tumor metastasis and OS among cancer patients [15, 16]. Thus, we hypothesized that lactate may be associated with the reprogramming and recruitment of TAMs.

Compared with the highly subjective and undynamic classical methods of cell behavior research, such as the tube formatting assay and the transwell invasion assay, microfluidic coculture chips may serve as more effective tools for the study of macrophage behavior because of their utility for quantitative and dynamic observation in the tumor microenvironment [17, 18]. Additionally, the chemokine gradients created by diffusion inside a microfluidic chip can better mimic the spatiotemporal bio-signals observed *in vivo* [17, 18]. Therefore, in the present work, we designed a microfluidic coculture chip and investigated the influence of lactate shuttling on the functional polarization and spatial distribution of cancer cells and macrophages.

## RESULTS

### Design of the microfluidic coculture chip

**Figure 1:**
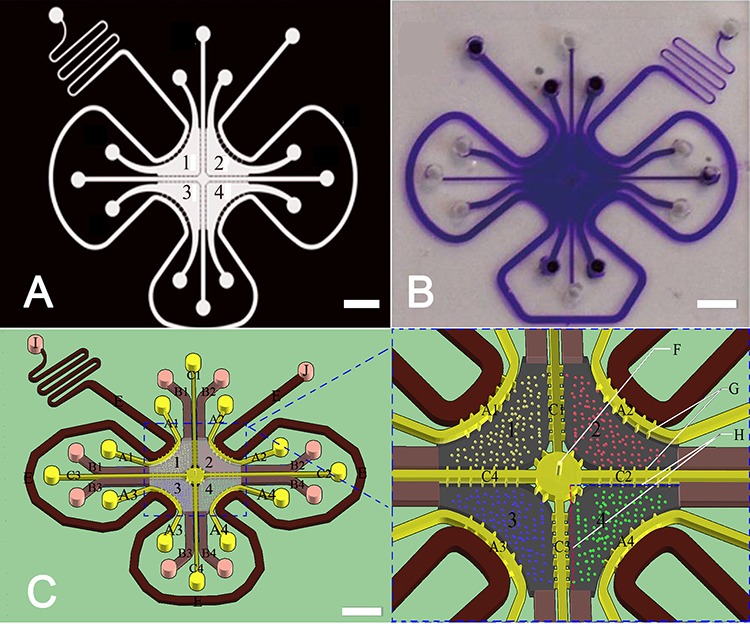
The microfluidic coculture chip and its design **A.** Flask mask was used to create the master plate. Scale bar: 3 mm. **B.** The fabricated microfluidic chip. Scale bar: 3 mm. **C.** Schematic of the microfluidic chips. The height of the chamber and the channels is 0.05 mm. A1, A2, A3, and A4 represent channels with a length of 8.6 mm and a width of 0.2 mm. B1, B2, B3, and B4 represent channels with a length of 5.4 mm and a width of 0.6 mm. C1, C2, C3, and C4 represent channels with a length of 8.8 mm and a width of 0.2 mm. G represents a channel with a length of 0.1 mm and a width of 0.05 mm. The length and the width of the migration channel between every two cell chambers are 0.4 mm and 60 μm, respectively. The length of line H is 3 mm. F represents the hole of perfusion glue with a diameter of 0.05 mm. Scale bar: 3 mm.

**Figure 2:**
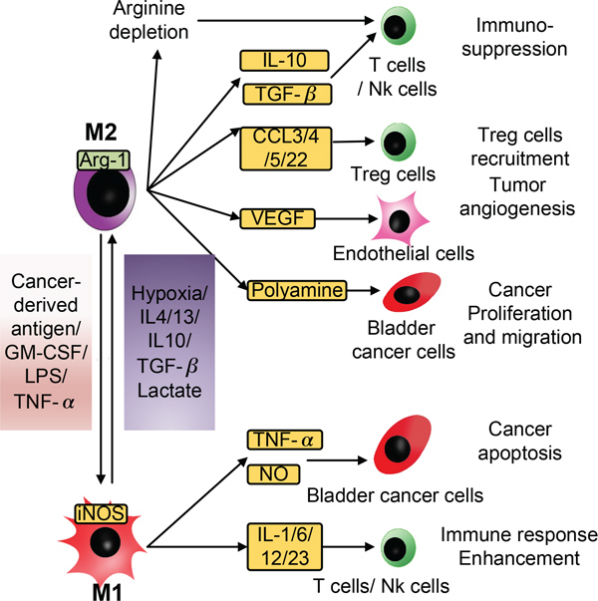
Schematic of the diverse effects of M1 and M2 macrophages on bladder cancer cells Macrophages are highly versatile immune cells that can exert anti- and pro-tumor effects at the same time. The M1/M2 model is usually used to interpret the complicated nature of macrophages. In response to stimulation by cancer-derived antigens, LPS, or TNF-α, TAMs become M1 macrophages, secrete NO and TNF-α to facilitate the apoptosis of cancer cells, and secrete IL-1, IL-6, IL-12, and IL-23 to enhance the immune response. When stimulated by hypoxia, IL-4, IL-13, IL-10, TGF-β, or lactate, TAMs polarize into M2 macrophages and cause immunosuppression, tumor angiogenesis, proliferation and migration by secreting a series of immune-regulating factors including IL-10, TGF-β, CCLs, VEGF, and polyamine.

**Figure 3:**
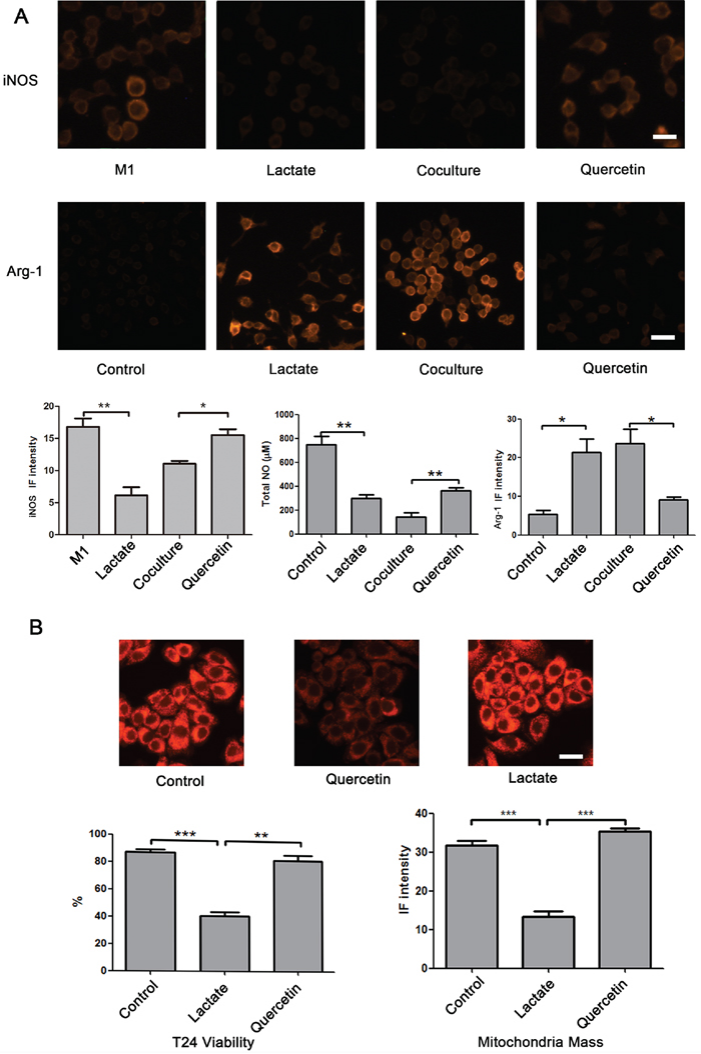
Effect of lactate shuttling on the re-education of macrophages by bladder cancer cells **A.** Lactate significantly inhibited the expression of iNOS induced by LPS and TNFα, and when M1 macrophages were cocultured with T24 cells, lactate blockade using quercetin augmented the expression of iNOS in M1 macrophages. Additionally, lactate remarkably reduced the secretion of NO and elevated the expression of Arg-1 in RAW264.7 cells. Alternatively, quercetin increased the secretion of NO and reduced the expression of Arg-1 in macrophages that were cocultured with T24 cells in microfluidic devices. RAW 264.7 cells were seeded into chambers 1 and 3. To assess the expression of iNOS, RAW264.7 cells were perfused with complete medium containing 100 ng/mL LPS and 100 U/mL IFNγ for the first 6 hour and were then perfused with complete medium from 6 to 48 h. Otherwise, RAW264.7 cells were cultured in complete medium. Chambers 2 and 4 were subjected to different treatments as follows: in the control group, chambers 2 and 4 were perfused with complete medium; in the lactate group, chambers 2 and 4 were perfused with complete medium containing 13 mM lactate; in the coculture group, T24 cells were cultured in chambers 2 and 4 in complete medium; and in the quercetin group, T24 cells were cultured in chambers 2 and 4 in complete medium containing 10 μM quercetin (Supplementary Table S1 experiment 1). **B.** Quercetin reduced the viability and the MitroTracker fluorescence intensity of T24 cells; however, after treatment with lactate, the values of these parameters were higher than those following treatment with quercetin alone. RAW264.7 cells or T24 cells were seeded into chambers 1 and 3 or 2 and 4, respectively. The cells were perfused with different media as follows: in the control group, the RAW264.7 and T24 cells were cultured in complete medium; in the quercetin group, the RAW264.7 cells were cultured in complete medium, and the T24 cells were cultured in complete medium containing 10 μM quercetin; and in the lactate group, the RAW264.7 cells were cultured in complete medium containing 13 mM lactate, and the T24 cells were cultured in complete medium containing 10 μM quercetin (Supplementary Table S1 experiment 2). Original magnification: × 400. *P < 0.05, **P < 0.01, ***P < 0.001. Scale bar: 10 μm.

**Figure 4:**
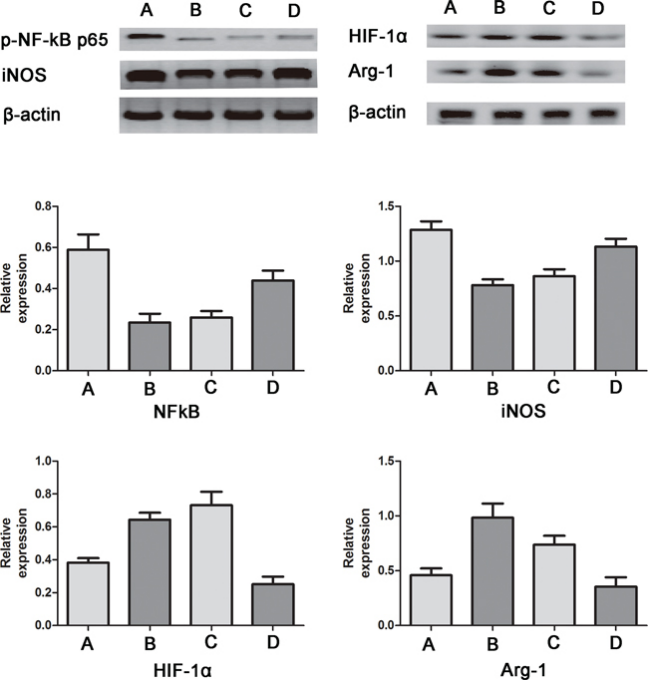
Western blotting for iNOS, p-NF-κB-p65, Arg-1 and HIF-1α in RAW264.7 cells **A.** control group; **B.** lactate group; **C.** coculture group; **D.** quercetin group. Compared with the control group, the expression of Arg-1 and HIF-1α was increased, and the expression of iNOS and p-NF-κB-p65 was decreased in the lactate group and the coculture group. Moreover, compared with the coculture group, the quercetin group showed increased expression of iNOS and p-NF-κB-p65 but reduced expression of Arg-1 and HIF-1α. The relative protein expression levels were calculated by dividing the grey value of the given protein by the grey value of β-actin.

To simulate the *in vivo* microenvironment of bladder cancer, we generated a microfluidic coculture chip using photolithography and soft-lithography techniques. This microfluidic chip consisted of four culture chambers, which could be seeded with cancer cells, macrophages or other cancer-related stromal cells (Figures 1, 2, 3 and 4). To observe the spatial distribution of cancer cells and macrophages, a Matrigel channel and 7 migration channels (length: 400 μm, width: 60 μm) were placed between every two adjacent culture chambers (Figure 1 C1, C2, C3 and C4). In this microfluidic device, if the duration of the test was sufficient, the cells could travel through the migration channel, resulting in the mixing of cells from different chambers. However, in our study, the test duration was less than 3 days, which is not long enough for the cells to migrate to other cell chambers; thus, the mixing of different cell types was impossible, and only the movement of proteins and reagents should be taken into consideration. Therefore, the influence of reagents on different cell types could be analyzed separately using a classical statistical method. To change and collect the culture medium in the culture chamber and to avoid the cell damage caused by shearing force, we designed a shearing force-free medium channel that was connected to culture chambers 1, 2, 3 and 4 via channels A1, A2, A3 and A4, respectively (Figure 1 channel E).

### Re-education of macrophages by bladder cancer cells

Macrophages are highly versatile immune cells that exert anti- and pro-tumor effects at the same time. M1 macrophages are known to act as anti-tumor cells due to their high expression of iNOS, nitric oxide, and inflammatory cytokines such as TNF-α and IL-1. M2 macrophages are known to function as inflammation-regulating and pro–angiogenic cells due to their elevated production of Arg-1 and VEGF. M2 macrophages are usually found in granulation tissue and play an important role in the wound-healing process. Cancer cells are considered to be capable of re-educating macrophages into the M2 phenotype (Figure 2) [19].

To test whether lactate can inhibit the polarization of M1 macrophages, LPS- and TNF-α-treated RAW264.7 cells, which are generally recognized as M1 macrophages, were seeded into chambers 1 and 3 and cultured in complete medium or complete medium supplemented with 13 mM lactate for 24 hours. Then, we measured the expression of iNOS using immunofluorescence (IF) staining and assessed the iNOS IF intensity using ImageJ software. The IF intensity of iNOS was significantly reduced after lactate treatment. (*p* < 0.01). Next, we cocultured M1 macrophages and T24 cells, which are a TCCB cell line, in our chip, and perfused the M1 macrophages with complete medium or complete medium supplemented with quercetin. The IF intensity of iNOS was reduced in the coculture group but was remarkably increased in the quercetin group (*P* < 0.01). These results showed that lactate shuttling inhibited the M1 polarization of macrophages and that blockade of the lactate shuttle prevented the re-education of tumor-associated macrophages by bladder cancer cells (Figure 3A).

To test the re-education function of lactate in M2 macrophages, we first seeded RAW264.7 cells, a mouse monocyte/macrophage cell line, into chambers 1 and 3 and cultured these cells in complete medium or complete medium supplemented with 13 mM lactate for 24 hours. Then, we measured the total NO concentration in the culture medium using a total NO assay kit and performed immunofluorescence staining for the Arg-1 protein from RAW264.7 cells. Compared with the control group, RAW264.7 cells of the lactate group showed a significant decrease in the total NO content (*P* < 0.01) but a remarkable increase in the levels of Arg-1 (*P* < 0.05) (Figure 3A). This result suggested that lactate reprogrammed macrophages into an M2 phenotype. Next, two groups were prepared to assess the role of lactate shuttling in the re-education of macrophages by bladder cancer cells. T24 cells or RAW264.7 cells were cultured in chambers 1 and 3 or 2 and 4, respectively. In the coculture group, the cells were cultured in complete medium, whereas in the quercetin group, the cells were cultured in complete medium containing 10 μM quercetin, which blocks the lactate shuttle by inhibiting monocarboxylic acid transporters [20]. The level of NO was elevated and the expression of Arg-1 protein was reduced in the RAW264.7 cells in the quercetin group compared with those in the coculture group (Figure 2A, *P* < 0.01 and *P* < 0.05, respectively). This result suggested that blockade of the lactate shuttle inhibited the re-education of macrophages by bladder cancer cells.

Furthermore, to precisely identify the influence of lactate shuttling, we used western blotting to assay the expression of Arg-1, hypoxia-inducible factor 1α (HIF-1α), iNOS and p-NF-κB-p65 from macrophages under different circumstances. However, the relatively low seeding capacity of this chip made it difficult to collect an adequate number of cells for western blotting. Therefore, as an alternative, 6-well plates were used for RAW264.7 cell monocultures, and transwell chambers were used for RAW264.7 cell cocultures. The results showed that lactate at a concentration of 13 mM increased the expression of Arg-1 and HIF-1α but reduced the expression of iNOS and p-NF-κB-p65. More importantly, quercetin increased the expression of iNOS and p-NF-κB-p65 but reduced the expression of Arg-1 and HIF-1α in RAW26.7 cells cocultured with T24 cells (Figure 4). These results were in agreement with the immunofluorescence staining results mentioned above. More importantly, bladder cancer cell-derived lactate was found to upregulate the HIF-1 pathway, creating a “pseudo-hypoxic” microenvironment.

Furthermore, to estimate the influence of lactate shuttling on bladder cancer cell viability, T24 cells were cocultured with RAW264.7 cells. T24 cells were perfused with complete medium (control group) or complete medium containing 10 μM quercetin (quercetin group and lactate group), and RAW264.7 cells were perfused with complete medium (control group and quercetin group) or complete medium containing 13 mM lactate (lactate group) (Supplementary Table S1 experiment 2). AO/EB and MitoTracker staining showed that compared with the control group, T24 cells of the quercetin group displayed significantly decreased viability and mitochondrial mass (*P* < 0.001). However, when RAW264.7 cells were treated with lactate, the viability and mitochondrial mass of T24 cells were higher than those following quercetin treatment alone (*P* < 0.01 and *P* < 0.001, respectively) (Figure 3B). This result implied that lactate shuttling reduced the apoptosis and mitophagy of bladder cancer cells in the tumor microenvironment.

### The gradient of signaling molecules in the culture chambers

**Figure 5:**
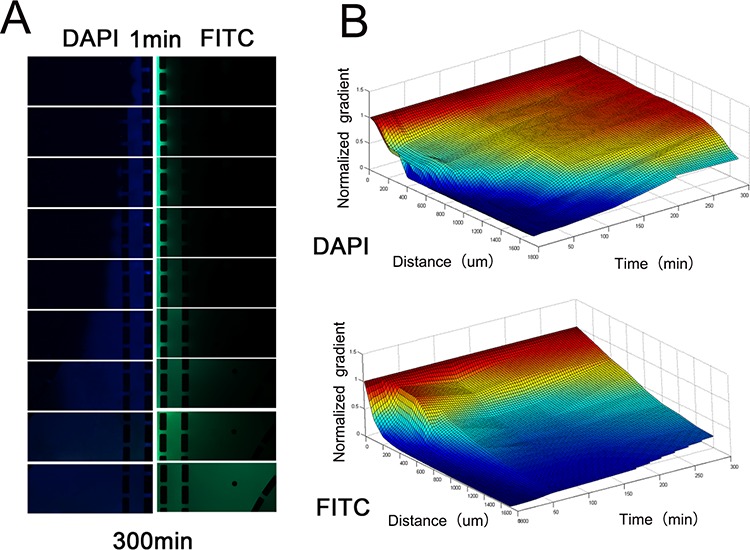
Concentration distribution of DAPI and FITC-dextran **A.** The images of blue (DAPI) and green fluorescence (FITC-dextran) in the Matrigel channels and culture chambers. Images were captured every 30 minutes. **B.** The fluorescence intensity was adjusted according to the fluorescence intensity of another microfluidic chip filled with 10 μg/ml DAPI and FITC-dextran dissolved in 5 mg/ml Matrigel to control for the influence of photobleaching. Simulation of the gradient distribution in the microfluidic chip was performed using Matlab software.

In the tumor microenvironment, vascular malformation is common, and the diffusion rate is a determinant of the concentration of signaling molecules, which plays an important role in the migration of cancer cells and the recruitment of macrophages. A relatively stable concentration gradient is one of the major advantages of microfluidic coculture chips. Next, we simulated the concentration distribution of signaling molecules with different molecular weights in the microfluidic chips using FITC-dextran and DAPI. Compared with FITC-dextran (MW: 17 kD), DAPI (MW: 277.3 D) showed a higher diffusion rate and more rapidly reached homeostasis in the microfluidic chips (Figure 5). Thus, small molecules diffuse faster and deeper than large signaling proteins in this microfluidic device.

### Recruitment of macrophages and the influence of macrophages on cancer metastasis

**Figure 6:**
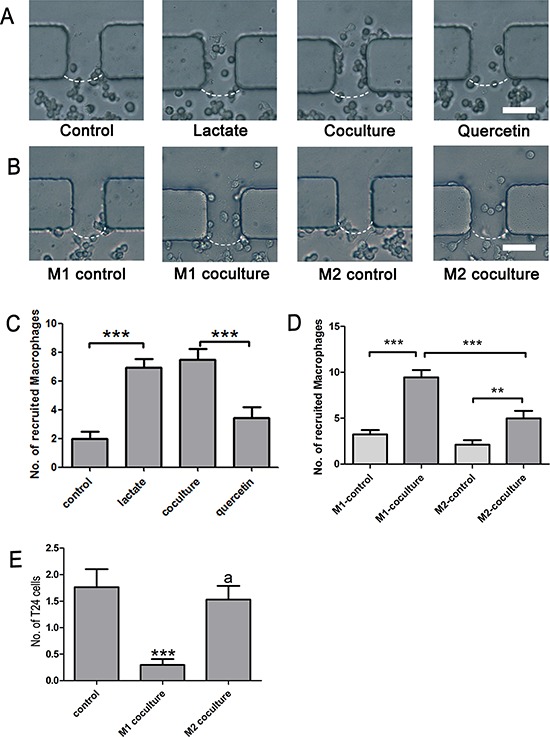
Recruitment of macrophages and metastasis of cancer cells **A.** and **C.** Lactate alone induced the migration of macrophages, and quercetin significantly inhibited the recruitment of macrophages by T24 cells; **B.** and **D.** T24 cells recruited M1 and M2 macrophages; **E.** M1, but not M2, macrophages reduced the motility of T24 cells. Original magnification: × 400. *P < 0.05, **P < 0.01, ***P < 0.001.

Cancer cells can recruit monocytes from blood via tumor-derived chemokines and antigens. This process, which may be the main reason for the high macrophage density in bladder cancer tissue, is important for the generation, development and metastasis of bladder cancer. To evaluate the role of lactate shuttling in the recruitment function of T24 cells, channels C1, C2, C3 and C4 were filled with Matrigel, and the number of RAW264.7 cells that migrated into the Matrigel was calculated. We first assessed the influence of lactate on macrophage migration. In the lactate group, the number of migrated qRAW264.7 cells was greater than that of the control group (*P* < 0.001). This result implied that lactate exerts a chemotactic effect on macrophages. Next, we assessed the influence of MCT blockade on the recruitment of macrophages by T24 cells, and the number of migrated RAW264.7 cells was smaller in the quercetin group than in the coculture group (*P* < 0.001) (Figure 6A, 6C). This result suggests that bladder cancer cells recruit macrophages via the lactate shuttle. In addition, we tested the difference in the numbers of migrated cells between M1 and M2 macrophages. The number of migrated RAW264.7 cells was significantly higher in the M1-coculture and M2-coculture groups than in the corresponding control groups (*P* < 0.001 and *P* < 0.01, respectively) (Figure 6B, 6D). Furthermore, compared with the M2-coculture group, the M1-coculture group displayed a greater number of migrated RAW264.7 cells (*P* < 0.001) (Figure 6B, 6D). This finding suggests that M1 macrophages are more likely to be recruited by these cancer cells.

Furthermore, we tested the influence of macrophages on the metastasis of T24 cells. The number of T24 cells that migrated to the Matrigel region was remarkably reduced when the cells were cocultured with M1 (*P* < 0.01), but not M2, macrophages (Figure 6E). This result suggested that classically activated macrophages inhibited the metastasis of bladder cancer cells but that alternatively activated macrophages do not exert a direct pro-metastatic effect on bladder cancer cells.

## DISCUSSION

It is generally agreed that the tumor microenvironment is too complicated to be reflected by traditional techniques. Thus, the utilization of microfluidic techniques for the study of cancer has been greatly promoted in recent years because this system enables precise control of experimental parameters, reproducible design of the tumor microenvironment, and real-time monitoring of experiments. Several microfluidic coculture chips have been established previously, but most of them have been designed with a focus on only one aspect of cancer cell-macrophage interactions, such as the migration of cancer cells or the secretion of products by macrophages. In the present work, we used a multi-channel microfluidic chip to simulate the microenvironment of bladder cancer. The functional status, migration viability and product concentration of the cells could be integrally tested using this chip. Therefore, we were able to more comprehensively evaluate the influence of lactate shuttling in the bladder cancer microenvironment.

Macrophages are one of the most important components of the tumor microenvironment. They are highly versatile immune cells that exert anti- and pro-tumor effects at the same time. The M1/M2 model is usually used to interpret the complicated nature of macrophages. M1 macrophages are pro-inflammatory cells, and M2 macrophages are anti-inflammatory and pro-angiogenic cells. Cancer cells are considered to be capable of re-educating macrophages into the M2 phenotype [19]. In particular, in patients with bladder cancer, macrophages are the major target of BCG therapy [6], and the re-education of macrophages may increase the density of M2 tumor-associated macrophages, which may lead to resistance to BCG immunotherapy [3]. Moreover, high M2 macrophage density was reported to be positively associated with tumor size, tumor stage, nodal metastasis, and histological grade [21]. Therefore, elucidating the mechanism underlying the re-education of TAMs is important for the treatment of bladder cancer. Our study revealed that the lactate shuttle between bladder cancer cells and TAMs may be a key mechanism of macrophage re-education. Lactate was found to significantly elevate the expression of Arg-1 and HIF-1α. Consistent with our results, Constant *et al*. [22] showed that lactate promoted VEGF synthesis in macrophages and subsequently induced angiogenesis. These results indicated that lactate can re-educate macrophages into an M2 phenotype. Further, we found that quercetin, an agent that blocks lactate shuttling by inhibiting the monocarboxylic acid transporter (MCT) [20], reduced Arg-1 and HIF-1α expression in macrophages. In addition, quercetin reduced the viability and mitochondrial mass of TCCB cells; however, after treatment with lactate, these parameters were restored to normal levels. These results further suggested that the re-education of macrophages by TCCB cells was highly dependent on cancer cell-TAM lactate flux.

**Figure 7:**
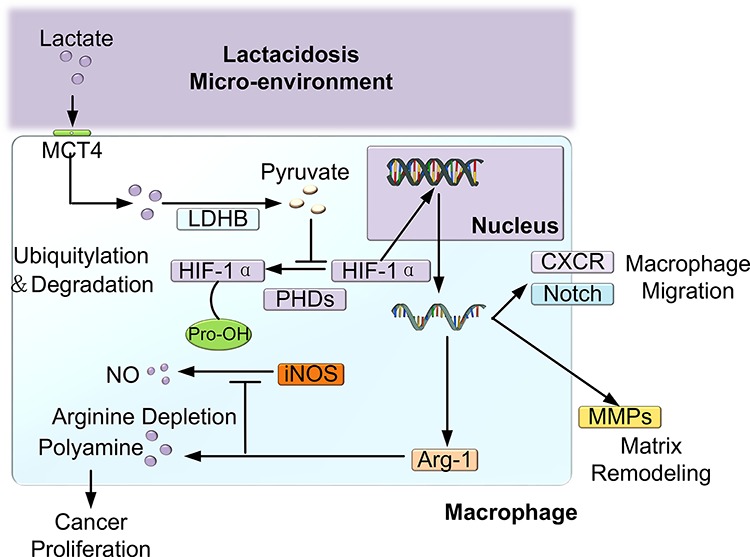
Schematic of the effect of lactate shuttling on the reprogramming and recruitment of macrophages Lactate was secreted into the tumor microenvironment via MCT4 and was then transported into macrophages via MCT1. Subsequently, lactate was metabolized to pyruvate by LDH1; then, pyruvate competitively inhibited the activity of PHDs, which prevented the ubiquitylation of hypoxia-inducible factor 1α (HIF-1α) and its destruction by the proteasome. Subsequently, HIF-1α may create “pseudo-hypoxia” by enhancing the transcription of a series of hypoxia-related genes, such as Arg-1, which metabolizes arginine to provide substrates for cancer cell proliferation. Additionally, HIF-1α may activate the transcription of the C-X-C chemokine receptor (CXCR) and Notch [25, 26], which are important signaling factors in cell migration. Furthermore, HIF-1α can promote matrix remodeling by increasing the secretion of MMPs.

It was well known that cancer cells are programmed to rely on aerobic glycolysis to support their proliferation and anabolic growth. As shown in Figure 5, elevated levels of lactate were secreted into the tumor microenvironment via MCT4, and then, the cancer cell-derived lactate was transported into macrophages via MCT1 [15]. Subsequently, lactate was metabolized to pyruvate by LDH1. Then, pyruvate competitively inhibited the interaction between α-ketoglutarate and prolyl hydroxylases (PHDs), which prevented the ubiquitylation of hypoxia-inducible factor 1α (HIF-1α) and its destruction by the proteasome [23]. Subsequently, HIF-1α may create “pseudo-hypoxia” by enhancing the transcription of a series of hypoxia-related genes such as Arg-1, which can metabolize arginine to provide the substrates for cancer cell proliferation [24]. HIF-1 may also activate the transcription of the C-X-C chemokine receptor (CXCR) and Notch [25, 26], which are important signaling factors in cell migration [27]. HIF-1 can also promote matrix remodeling by increasing the secretion of MMPs [28] (Figure 7).

In addition, we found that lactate inhibited the expression of iNOS and the secretion of nitric oxide by M1 macrophages and downregulated the NF-κB pathway, which was also blocked by quercetin. iNOS is the most efficient nitric oxide synthase found in human cells. When stimulated by endogenous or exogenous antigens, macrophages express high levels of iNOS and produce great quantities of NO. Further, NO is one of the most important functional endogenous molecules in cancer-related inflammation. NO competes with oxygen for cytochrome c oxidase to cause mitochondrial dysfunction [29]. NO can also induce the autophagic destruction of mitochondria, termed mitophagy [20]. Moreover, cytochrome C from mitochondria can be released into the cytosol, bind to apoptotic protease-activating factor 1, and activate execution caspases, which ultimately catalyzes the fragmentation of DNA and the formation of apoptotic bodies [30]. The NF-κB pathway plays the most important role in cancer-related inflammation by mediating the DNA transcription of a series of inflammation-related cytokines such as IL-1β. Additionally, the NF-κB pathway is essential for the transcription of iNOS [31]. These results suggest that lactate shuttling inhibits the M1 polarization of tumor-associated macrophages by down-regulating the NF-κB pathway and reducing the secretion of NO and inflammation-related cytokines. These events greatly reduce the intensity of the immune response and facilitate the survival of bladder cancer cells.

In addition, we found that DAPI diffused faster and deeper than FITC-dextran in the microfluidic tumor microenvironment model. This finding is in agreement with the findings that diffusivity decreased monotonically with the MW of the solute in all ECM gels, as reported by Roland et.al [32]. The flexibility, hydrophobicity and charge distribution of the solute molecule can influence the solute diffusion rate in polymer systems [33]. The MW of lactate is only one-thousandth that of signaling proteins. Furthermore, lactate is only composed of C-C bonds, and this structure could provide lactate molecules with great flexibility. Alternatively, signaling proteins consist of many amino acids, which are rich in conjugated double bonds and benzene ring structures; thus, these proteins have limited molecular flexibility. More importantly, many proteins, such as MCP-1 and VEGF, contain motifs that are specific for components of the extracellular matrix and that can bind to the extracellular matrix, such binding could greatly hinder the diffusion of these signaling proteins [34, 35]. In contrast, no reports have shown that lactate can bind to the extracellular matrix, and lactate is highly hydrophilic and electroneutral in the acidic tumor microenvironment. Therefore, these signaling proteins diffuse much slower than lactate in the tumor microenvironment. TNF-α, TGF-β and CCL2 were recognized as the major drivers of the polarization and chemotaxis of macrophages [17, 36]. However, TAMs are usually localized to the high perfusion region of tumor tissue, such as the invasive edge and perivascular areas [37]. Therefore, the diffusion rate may notably influence the concentrations of cytokines in the TAM-rich area, and small molecules may play more important roles than cytokines in the cancer-induced re-education of TAMs.

Furthermore, our study showed that TCCB cells recruited M1 and M2 macrophages. Although lactate was previously found to be capable of promoting the migration of cancer cells [38] and endothelial cells [39], its chemotactic effect on macrophages has not yet been reported. Notably, for the first time, we found that lactate alone induced the migration of macrophages and that quercetin significantly inhibited the recruitment of macrophages by TCCB cells. The mechanism underlying this activity might be that the stabilized HIF-1α bound to the promoter of genes encoding C-X-C chemokine receptor 4 (CXCR4), matrix metalloproteinases (MMPs) or Notch, leading to the enhanced expression of these proteins, which are closely associated with cell mobility [28, 40] (Figure 7). Additionally, lactate was converted to pyruvate in the cytoplasm of macrophages and then entered the tricarboxylic acid cycle or served as a substrate for biosynthesis. Thus, lactate may also serve as an alternate nutrition source to support macrophages, ultimately leading to the migration of macrophages along the nutrient gradient. We speculate that the lactate-induced recruitment of macrophages may be an important source of TAMs in cancer tissue, although the detailed mechanism underlying this effect should be investigated further. In addition, we found that M1 macrophages reduced the motility of TCCB cells. This result suggested that M1 macrophages strongly inhibited the metastasis of TCCB cells and that TCCB cells could escape from the inhibition of metastasis caused by the immune response by re-educating macrophages into the M2 phenotype. This result was in concert with the clinical findings that immunopotentiator treatment, such as intravesical BCG instillation, extensively induced the M1 polarization of TAMs and subsequently prevented the progression and metastasis of TCCB [41]. Further research is necessary to evaluate the validity and sensitivity of lactate shuttling blockade in clinical applications.

Although M2 macrophages were known to promote tumor metastasis by secreting TGF-β, EGF, FGF and VEGF [42], our study showed that M2 macrophages did not exert a significant pro-metastatic effect. This result suggested that TAMs did not exert a strong pro-metastatic effect on TCCB cells but that these cells might promote the metastasis of TCCB cells by inducing angiogenesis and lymphangiogenesis, as well as by creating a pro-tumor microenvironment at the site of metastasis [42]. Compared with the regional migration of cancer cells, the transvascular and lymphatic metastasis of TCCB cells are more complicated processes, and we will modify this microchip device according to an established micro-vascular model [43] and other immune-related microfluidic chips [44] in a subsequent study.

## MATERIALS AND METHODS

### Fabrication of the microfluidic coculture chips

Photolithography and soft-lithography techniques were utilized to fabricate the microfluidic coculture device. A schematic of the microfluidic coculture chips is shown in Figure 1. Briefly, a transparent mask was created using automated computer-aided design software and was printed by a high-resolution printer. Silicon wafers coated with 100 μm-thick SU-8 were covered by the mask and were prepared by photolithography. Polydimethylsiloxane (PDMS) mixed with the curing agent (1:10) was poured on the master plate, which was then cured in an 80°C oven for 2 h. Subsequently, the polymerized PDMS was peeled from the master plate, and inlets were created using a 1 mm puncher. A glass slide was treated with boiling 75% sulfuric acid for 30 min and then washed with distilled water. The PDMS and the glass slide were treated with oxygen plasma for 30 s to form a covalent bond between them. The microfluidic coculture chips were baked in an 80°C oven overnight. Ultimately, this microfluidic chip consisted of four culture chambers, 28 migration channels, and a medium channel. Every culture chamber was connected to the adjacent culture chambers by 7 migration channels. Up to four different types of cells could be seeded into the culture chambers. The migration of the cells could be measured in the migration channel.

### Cell culture

The mouse monocyte/macrophage cell line RAW 264.7 and the human bladder transitional cell carcinoma cell line T24 were obtained from the Central Laboratory of the Affiliated Hospital of Qingdao University. Unless specifically mentioned, RAW 264.7 and T24 cells were cultured in Roswell Park Memorial Institute (RPMI) 1640 medium (Hyclone, Logan, UT, USA) supplemented with 10% fetal bovine serum (FBS, Hyclone) and 1% pen-strep-ampho antibiotic (Hyclone) at 37°C in a 75% humidity incubator.

### Effect of lactate shuttling on the re-education of macrophages by bladder cancer cells

RAW 264.7 and T24 cells were separately harvested by scraping and then centrifuged at 800 rpm for 5 min. The density of both cell types was adjusted to 5 × 106 cells/mL. We first measured the amount of lactate secreted by T24 cells into the conditioned medium. Because we found that the lactate secretion rate was 13 mM/24 h, lactate was applied at a concentration of 13 mM. To study the re-education of macrophages, RAW 264.7 cells were seeded into chambers 1 and 3 and cultured in complete medium. Then, chambers 2 and 4 were used for different treatments as follows: in the control group, chambers 2 and 4 were perfused with complete medium; in the lactate group, chambers 2 and 4 were perfused with complete medium containing 13 mM lactate (Sigma, Louis, MO, USA); in the coculture group, T24 cells were cultured in chambers 2 and 4 in complete medium; and in the quercetin group, T24 cells were cultured in chambers 2 and 4 in complete medium containing 10 μM quercetin (Solarbio, Beijing, China) (Supplementary Table S1 experiment 1). To estimate the influence of lactate shuttle-mediated re-education of macrophages on bladder cancer cell viability, RAW264.7 cells or T24 cells were seeded into chambers 1 and 3 or 2 and 4, respectively. The cells were perfused with different media as follows: in the control group, the RAW264.7 and T24 cells were cultured in complete medium; in the quercetin group, the RAW264.7 cells were cultured in complete medium, and the T24 cells were cultured in complete medium containing 10 μM quercetin; and in the lactate group, the RAW264.7 cells were cultured in complete medium containing 13 mM lactate, and the T24 cells were cultured in complete medium containing 10 μM quercetin. The media in this microfluidic device were replaced every 6 hours. After cell seeding for 36 h, the activation status of macrophages was identified based on the total nitric oxide (NO) content and the Arg-1 expression level. Cell viability was assessed by AO/EB and MitoTracker staining (Supplementary Table S1 experiment 2).

### Detection of total NO content

The total NO content in the culture medium from chambers 2 and 4 was measured using a total NO assay kit (Beyotime Biotechnologies, Shanghai, China) according to standard procedures. Briefly, standard nitrite solutions were used to prepare a standard curve. Samples were treated with nitrate reductase for 40 min and then reacted with Griess reagent for 10 min at room temperature. The absorbance at 540 nm was measured, and the total NO content was determined according to the standard curve.

### Cell immunofluorescence and immunoblotting

Cells in chambers 2 and 4 were fixed in 4% paraformaldehyde for 30 min and then blocked using normal goat serum at 37°C for 30 min. Subsequently, the samples were incubated in an anti-Arg-1 antibody (ABclonal) at 4°C overnight. After washing with PBS, the samples were incubated in a fluorescently labeled secondary antibody at room temperature for 1 hour. Finally, images were acquired using an inverted fluorescence microscope (Olympus, Japan), and the immunofluorescence (IF) intensity of all images was determined using ImageJ software (v1.48).

Transwell cell culture inserts (Corning #3413) were used to coculture T24 cells with RAW264.7 cells. T24 cells were cultured in the upper compartment, and RAW264.7 cells were cultured in the lower compartment. Four groups were designed, and the culture medium was the same as that used for chambers 2 and 4 in the microfluidic chips (Supplementary Table S1 experiment 1). Additionally, for iNOS and NF-κB immunoblotting, the RAW264.6 cells were pretreated with 100 ng/mL LPS and 100 U/mL IFN-γ for 6 hours before culturing the cells in the transwell cell culture inserts. Anti-Arg-1 (ABclonal), anti-iNOS (CST), anti-NF-κB (CST) and anti-HIF-1α antibodies (CST) were used to detect the expression of Arg-1, iNOS, NF-κB, and HIF-1α, respectively, in RAW264.7 cells.

### Cell viability evaluation

The viability of cells in chambers 1 and 3 was evaluated using an acridine orange/ethidium bromide (AO/EB) double fluorescence kit (Solarbio). The cells in chambers 2 and 4 were incubated in AO/EB solution (1:1) for 2–5 min, and the number of viable cells was counted under an inverted fluorescence microscope (Olympus). In addition, the cells were incubated in MitoTracker dye (Invitrogen, Carlsbad, CA, USA) for 15 min, and the viability of cells was determined based on the mitochondrial mass.

### Concentration distributions of lactate and large signaling proteins in the culture chambers

After Matrigel polymerization, the concentration distribution in the culture chambers was measured with 10 ug/ml 4′,6-diamidino-2-phenylindole (DAPI, Solarbio) and 20 mg/ml fluorescein isothiocyanate (FITC)-dextran (Solarbio). The microfluidic device was placed in a 37°C incubator, and the culture medium was replaced every 15 minutes. Images were captured every 30 minutes for 20 hours. Additionally, another microfluidic chip was filled with 10 μg/ml DAPI and FITC-dextran dissolved in 5 mg/ml Matrigel. This chip was imaged every 30 minutes to control for the influence of photobleaching.

### Recruitment of macrophages and metastasis of cancer cells

RAW 264.7 cells were pretreated with 100 ng/mL lipopolysaccharide (LPS, Santa Cruz Biotechnology, Santa Cruz, USA) and 100 U/mL interferon-γ (IFN-γ, Peprotech Inc., Rocky Hill, NJ, USA) for 24 h to obtain M1 cells. Alternatively, RAW 264.7 cells were pretreated with 10 ng/ml IL-4 to obtain M2 cells. To mimic the recruitment of macrophages by bladder cancer cells, 4 groups of experiments were designed as follows: M1 control group (M1 macrophages cultured in chambers 2 and 4), M1 coculture group (T24 cells cultured in chambers 1 and 3 and M1 macrophages cultured in chambers 2 and 4), M2 control group (M2 macrophages cultured in chambers 2 and 4), and M2 coculture group (T24 cells cultured in chambers 1 and 3 and M2 macrophages cultured in chambers 2 and 4) (Supplementary Table S1 experiment 3). To evaluate the effect of macrophages on cancer cell metastasis, T24 cells were seeded into chamber 2, and untreated RAW 264.7 cells (control group), M1 macrophages (M1 coculture group), or M2 macrophages (M2 coculture group) were cultured in chamber 4. All of the chambers seeded with cells were perfused with RPMI 1640 medium supplemented with 1% FBS. RPMI 1640 medium supplemented with 15% FBS was perfused into chamber 1 (Supplementary Table S1 experiment 4). The number of migrated cells and the depth of invasion was measured after 48 h using a light microscope.

### Statistical analysis

Statistical analysis was performed using SPSS 12.0 statistical analysis software (SPSS Inc., Chicago, IL, USA). The data were analyzed using the paired *t*-test. Avalue of *P* < 0.05 was considered significant, and *P* < 0.01 was considered highly significant.

## CONCLUSIONS

In the present study, we used a microfluidic coculture chip to study the cancer cell-macrophage interactions in the bladder cancer microenvironment. We confirmed that TCCB cells reprogrammed macrophages into an M2 phenotype in a manner that depended on cancer cell-TAM lactate flux. Therefore, the lactate shuttle is one of the major causes of immunosuppression within the TCCB microenvironment, and MCTs may be a new treatment target for TCCB. Furthermore, the lactate shuttle may be a determinant of the density of TAMs in tumor tissue, although further research is necessary to elucidate the mechanism underlying the chemotactic effect of lactate shuttling.

## SUPPLMENTARY TABLE


