# Supplementary material for: Bladder cancer cells re-educate TAMs through lactate shuttling in the microfluidic cancer microenvironment
Source: Oncotarget. 2015 Oct 13;6(36):39196–210. doi: 10.18632/oncotarget.5538 (PMC4770766; doi:10.18632/oncotarget.5538)
Supplement: Supplementary file 1 [file oncotarget-06-39196-s001.pdf]

SUPPLMENTARY TABLE

Supplementary Table S1: Cell lines and medium in the microfluidic chips

Experiment	Group	Chamber 1		Chamber 3		Chamber 2 and 4	
		Cell	Medium	Cell	Medium	Cell	Medium
1	control	RAW264.7	complete medium[a]	RAW264.7	complete medium	No cell	complete medium
	lactate	RAW264.7	complete medium[a]	RAW264.7	complete medium	No cell	complete medium containing 13 mM lactate
	coculture	RAW264.7	complete medium[a]	RAW264.7	complete medium	T24	complete medium
	quercetin	RAW264.7	complete medium[a]	RAW264.7	complete medium	T24	complete medium containing 10 µM quercetin
2	control	RAW264.7	complete medium	RAW264.7	complete medium	T24	complete medium
	quercetin	RAW264.7	complete medium	RAW264.7	complete medium	T24	complete medium containing 10 µM quercetin
	lactate	RAW264.7	complete medium containing 13 mM lactate	RAW264.7	complete medium containing 13 mM lactate	T24	complete medium containing 10 µM quercetin
3	M1 control	No cell	complete medium	No cell	complete medium	M1 macrophage[b]	complete medium
	M1 coculture	T24	complete medium	T24	complete medium	M1 macrophage[b]	complete medium
	M2 control	No cell	complete medium	No cell	complete medium	M2 macrophage[b]	complete medium
	M2 coculture	T24	complete medium	T24	complete medium	M2 macrophage[b]	complete medium
4	control	No cell	RPMI1640 supplemented with 15% FBS	T24	RPMI1640 supplemented with 1% FBS	RAW264.7	RPMI1640 supplemented with 1% FBS
	M1 coculture	No cell	RPMI1640 supplemented with 15% FBS	T24	RPMI1640 supplemented with 1% FBS	M1 macrophage[b]	RPMI1640 supplemented with 1% FBS
	M2 coculture	No cell	RPMI1640 supplemented with 15% FBS	T24	RPMI1640 supplemented with 1% FBS	M2 macrophage[b]	RPMI1640 supplemented with 1% FBS

[a]When the expression of iNOS and p-NFkB-p65 was tested, RAW264.7 cells were perfused with complete medium with 100 ng/mL LPS and 100 U/mL IFN-γ in the first 6 hour, and then perfused with complete medium from 6 to 48 h.

[b]RAW 264.7 cells were pretreated with 100 ng/mL lipopolysaccharide and 100 U/mL interferon-γ for 24 h to obtain M1 cells, as well as pretreated with 10 ng/ml IL-4 to obtain M2 cells.
